# Multi-scale Jones polynomial and persistent Jones polynomial for knot data analysis

**DOI:** 10.3934/math.2025068

**Published:** 2025-01-22

**Authors:** Ruzhi Song, Fengling Li, Jie Wu, Fengchun Lei, Guo-Wei Wei

**Affiliations:** 1 School of Mathematical Sciences, Dalian University of Technology, Dalian 116024, Liaoning, China; 2 School of Mathematical Sciences, Hebei Normal University, Shijiazhuang 050024, Hebei, China; 3 Beijing Institute of Mathematical Sciences and Applications, Beijing 101408, China; 4 Department of Mathematics, Michigan State University, 426 Auditorium Road, East Lansing, MI 48824, USA; 5 Department of Biochemistry and Molecular Biology, Michigan State University, 426 Auditorium Road, East Lansing, MI 48824, USA; 6 Department of Electrical and Computer Engineering, Michigan State University, 426 Auditorium Road, East Lansing, MI 48824, USA

**Keywords:** knot data analysis, curve data analysis, Jones polynomial, localization, stability, protein flexibility, 57K10, 92C10

## Abstract

Many structures in science, engineering, and art can be viewed as curves in 3-space. The entanglement of these curves plays a crucial role in determining the functionality and physical properties of materials. Many concepts in knot theory provide theoretical tools to explore the complexity and entanglement of curves in 3-space. However, classical knot theory focuses on global topological properties and lacks the consideration of local structural information, which is critical in practical applications. In this work, two localized models based on the Jones polynomial were proposed, namely, the multi-scale Jones polynomial and the persistent Jones polynomial. The stability of these models, especially the insensitivity of the multi-scale and persistent Jones polynomial models to small perturbations in curve collections, was analyzed, thus ensuring their robustness for real-world applications.

## 1. Introduction

Knot theory, a branch of mathematics that focuses on the study of mathematical knots, is primarily concerned with classifying and analyzing knots based on their essential properties under ambient isotopy [1]. This approach allows mathematicians to disregard the specific manner in which knots are embedded in 3-space, emphasizing instead the invariants that remain unchanged under continuous deformations. There are several knot invariants, such as the knot crossing number, the knot group [1], the Alexander polynomial [2], the Jones polynomial [3], the knot Floer homology [4], and the Khovanov homology [5].

Knot theory has applications in many fields, including physics [6], chemistry [7], and biology [8–10]. In practical applications, however, two major challenges arise: Many structures do not form closed loops, and ambient isotopy can significantly alter local structures while preserving global knot characteristics. For example, open curves in the 3-space, such as polymers [11, 12], textiles [13], chemical compounds [14], and biological molecules [15, 16], often exhibit local entanglement that critically affects their physical properties and functions. Topological invariants, functions that remain unchanged under ambient isotopy, are essential for analysis of knots and links [17–19]. However, these invariants do not extend to open curves, since open curves can be continuously deformed without requiring cutting or rejoining, making topological equivalence inapplicable.

In recent years, methods that incorporate classical concepts from knot theory and are more applicable to practical problems have been proposed. Compared to topological data analysis (TDA), the concept of knot data analysis (KDA) was formally introduced in [20]. Panagiotou and Plaxco [21] demonstrated the utility of the Gauss link integral in protein entanglement, particularly to understand protein folding kinetics and improve future folding models. Based on this, Baldwin and Panagiotou [22] introduced a new measure of local topological and geometrical free energy based on writhing and torsion of protein chains, highlighting its critical role in the rate-limiting steps of protein folding. In addition, Baldwin et al. [23] extended these topological concepts to the study of the Severe acute respiratory syndrome coronavirus 2 (SARS-CoV-2) spike protein, showing how local geometric features such as writhe and torsion influence its stability and behavior. In a related effort, Shen et al. [20] introduced multi-scale Gauss link integral (mGLI), a novel method leveraging the Gauss link integral to quantify the entanglement and topological complexity of both open and closed curves at various scales. This versatile approach has broad applications in the analysis of curve structures in both physical and biological systems.

The Jones polynomial [3], a fundamental invariant in knot theory, provides a polynomial measure of entanglement that distinguishes different types of knots by smoothing their crossings. Panagiotou and Kauffman [24] extended this concept to an open curve and proposed a continuous measure of entanglement that converges to the classical Jones polynomial as the end points of an open curve approach each other. Barkataki and Panagiotou [25] further refined this by introducing the Jones polynomial for collections of curves, averaged in all projection directions. Building on these topological frameworks, Panagiotou and Kauffman [26] also used Vassiliev invariants to quantify the complexity of open and closed curves in 3-space. Furthermore, Wang and Panagiotou [27] explored the correlations between protein folding rates and topological measures, specifically writhe, average crossing number (ACN), and the second Vassiliev invariant, to understand the behavior of native protein states. In addition, Herschberg, Pifer, and Panagiotou [28] developed a computational tool that quantifies topological complexity in systems such as polymers, proteins, and periodic structures.

Using the Jones polynomial framework for the collections of disjoint open or closed curves proposed by Barkataki and Panagiotou in [25], this manuscript introduces two novel models: The multi-scale Jones polynomial, represented by a characteristic matrix, as described in Section 3.1, and the persistent Jones polynomial, represented by weighted persistent barcodes or weighted persistent diagrams, as described in Section 3.2. The weighted barcode was introduced by Cang and Wei in [29]. Both models have the ability to capture local and global entanglement properties in open or closed curve structures in the 3-space, thus, effectively representing their topological characteristics. These models provide an improved approach to solving problems in physical, biological, and chemical environments.

The stability of the proposed models is a key feature that ensures their robust applicability to real-world scenarios. Stability in this context refers to the robustness of persistent Jones polynomial models, including the multi-scale process, against small perturbations in the data. Minor changes in the positions or configurations of the curve segments should result in correspondingly minor variations in the computed measures. This property is essential for reliable analysis in noisy environments or datasets subject to slight distortions, such as those often encountered in physical, biological, and chemical systems. The models provide a reliable framework for characterizing the topological and geometric properties of complex structures.

The proposed models are applied to the prediction of B factors and the analysis of protein α-helix and β-sheet structures. The B factor, or Debye-Waller factor, is a critical metric in structural biology that represents the atomic displacement and flexibility within a protein structure and, thus, serves as an indicator of protein dynamics and stability. Traditional methods for predicting B factors have had limitations in capturing the topological information inherent in protein structures. To address this, we apply the multi-scale Jones polynomial model to the prediction of B factors, and achieve prediction accuracies of 0.899, 0.808, and 0.720 for small, medium, and large protein sets [30], respectively. Our results on these three datasets outperformed previous methods. In addition, the persistent Jones polynomial model is utilized to explore the structural properties of protein α-helix and β-sheet segments, with visual representations provided by barcodes that highlight the entanglement features across these secondary structures. The proposed multi-scale Jones polynomial and persistent Jones polynomial models have potential for curve data analysis (CDA).

The article is organized as follows: In Section 2, the fundamental construction of the Jones polynomial is introduced for the collections of curves in the 3-space. In Section 3, two new models of the Jones polynomial of curves in 3-space are established, namely, the multi-scale Jones polynomial (discussed in Section 3.1) and the persistent Jones polynomial (described in Section 3.2). In Section 4, the stability of these two local models is demonstrated. Section 5 presents applications of the new models, including the prediction of B factors and the exploration of α-helix and β-sheet structures. In Section 6, a discussion is presented on the selection of segmentation, localization, stability issues, and the comparison with classical persistent homology.

## 2. The Jones polynomial of curves in 3-space

The Jones polynomial [3] is an important invariant in classical knot theory, recognized for its ability to characterize the entanglement properties of knots and links. However, it is less applicable to practical scenarios, which often involve open curves in 3-space rather than closed curves. To address this limitation, Barkataki and Panagiotou [25] extended the concept to the collections of disjoint open or closed curves by defining a normalized version of the bracket polynomial, averaged over all projection directions. This adaptation increases its relevance to real-world applications. Moreover, the Jones polynomial for collections of disjoint open or closed curves converges to the classical Jones polynomial as the endpoints of these curves approach each other. When projected onto a 2-dimensional plane, a collection of curves in 3-space forms a linkoid, which can be generalized to multi-component knotoids describing open-ended knot diagrams. The theory of knotoids was first introduced by Turaev [31], with further developments on linkoids elaborated in [32–35].

### 2.1. Segment cycles

Before defining the bracket polynomial of linkoids, it is essential to introduce the concept of segment cycles associated with a state. Let L be a linkoid diagram consisting of multiple components. Let G={1,2,3,…,2n} denote the set of all endpoints (heads and legs) of L. A component of a linkoid with n components is represented as $l_{2j-1,2j}$, where j∈{1,2,…,n}. The head-leg pairing forms a product of n disjoint 2-cycles, denoted by $\overset{ˆ}{L}=(1,2)(3,4)\ldots (2n-1,2n)$.

Let S be a state corresponding to a choice of smoothing over all crossing points in L. This state induces a pairing represented by the product of n disjoint 2-cycles,

$$\overset{ˆ}{S}=\left(s_{1},s_{2}\right)\left(s_{3},s_{4}\right)\ldots \left(s_{2n-1},s_{2n}\right),$$

where each $s_{i}\in G$ and each pair $\left(s_{2j-1},s_{2j}\right)$ for j∈{1,2,…,n} represent the endpoints of a component in the state S.

For any endpoint a∈G, the set

$${\text{Orb}}_{S}(a)=\left\{x\in G|x=(\overset{ˆ}{L}\circ \overset{ˆ}{S}{)}^{m}(a),m\in Z\right\}$$

is defined as the *orbit* under the composition function $\overset{ˆ}{L}\circ \overset{ˆ}{S}$. The *segment cycle* of an endpoint a∈G is given by

$$\text{Seg}(a)={\text{Orb}}_{S}(a)⊔{\text{Orb}}_{S}(\overset{ˆ}{L}(a)).$$

It is notable that for any point $a\in G,\overset{ˆ}{L}(a)$ also belongs to the same segment cycle. Thus, a segment cycle always contains an even number of elements.

**Lemma 2.1.** [25, *Proposition 3.1] The number of segment cycles in a state* S, *denoted by* $|S{|}_{cyc}$, *satisfies* $1⩽|S{|}_{cyc}⩽n$.

**Figure 1.**
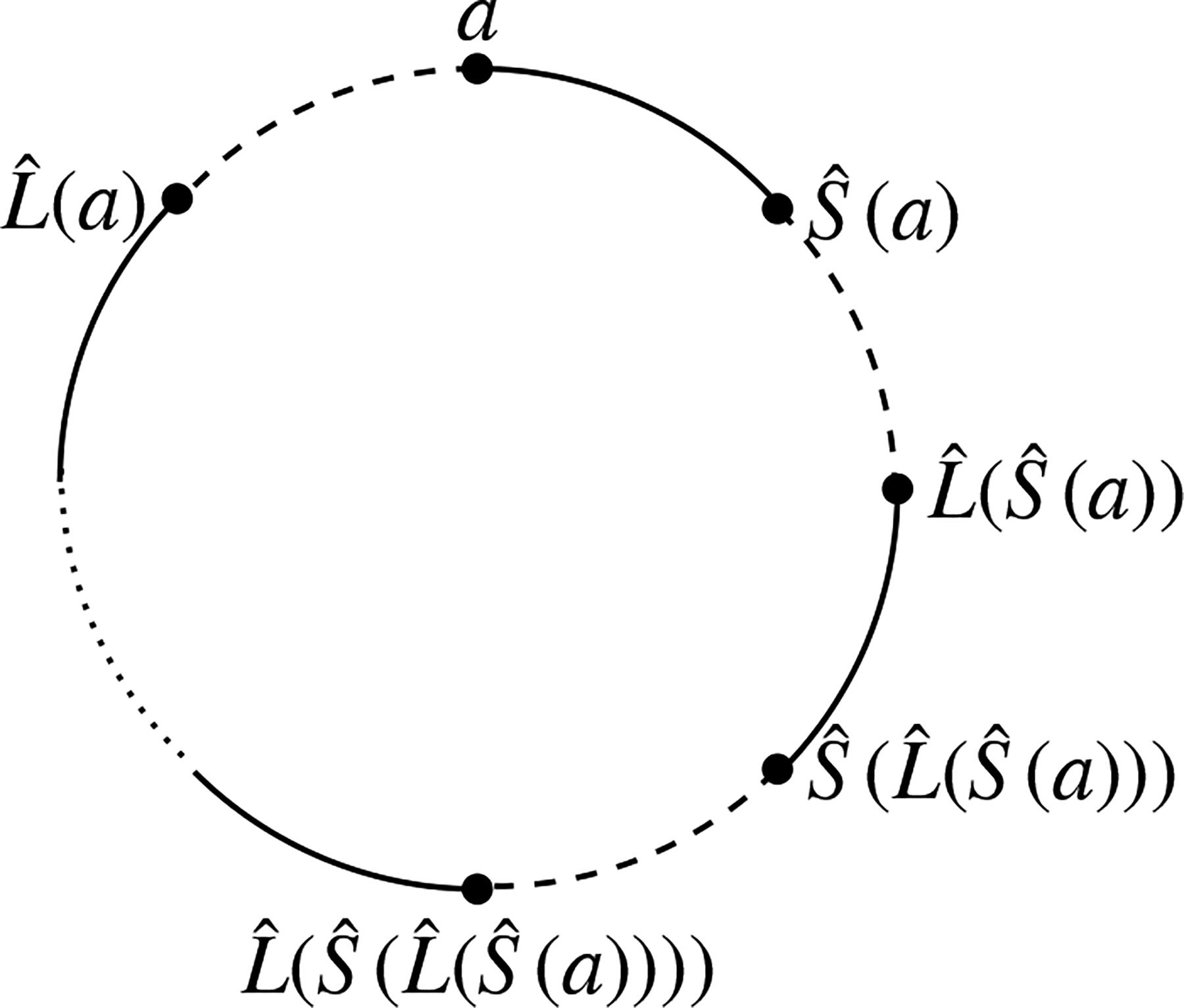
Representation of the segment cycle of a∈G.

Consider a state S of L with the associated pairing $\overset{ˆ}{S}$. Let Seg(a) be a segment cycle in S with |Seg(a)|=2k. This segment cycle can be represented by a circle marked with the 2k endpoints of L (see Figure 1). Let a∈G be the starting point of the circle. The remaining 2k-1 endpoints are uniquely sequenced in the circle as $\overset{ˆ}{S}(a),\overset{ˆ}{L}(\overset{ˆ}{S}(a)),\overset{ˆ}{S}(\overset{ˆ}{L}(\overset{ˆ}{S}(a)))$, etc., up to $\overset{ˆ}{L}(a)$. It is important to note that the arcs connecting adjacent points in this circular representation alternate between the functions $\overset{ˆ}{S}$ and $\overset{ˆ}{L}$. The points connected by $\overset{ˆ}{S}$ belong to the same component in state S, while the points connected by $\overset{ˆ}{L}$ belong to the same component in L.

**Figure 2.**
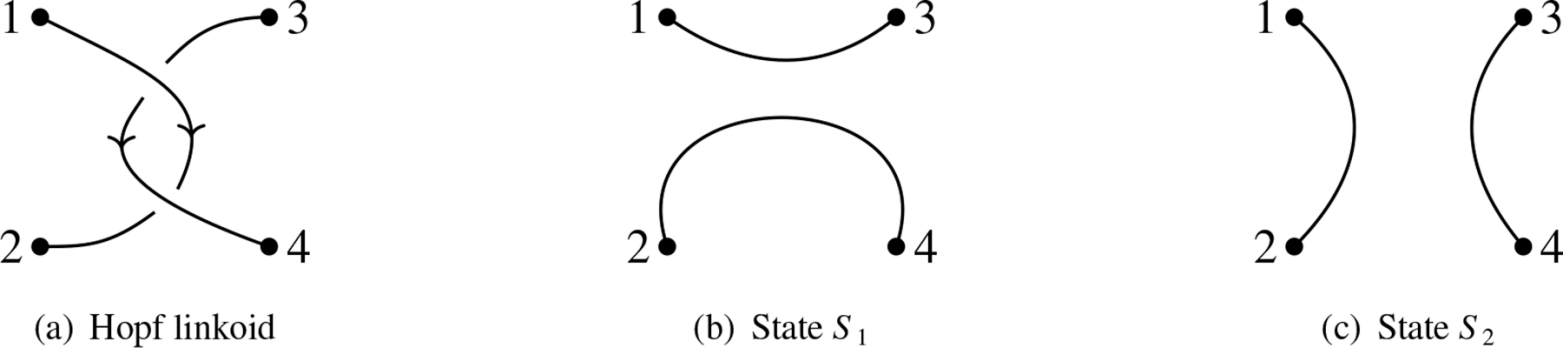
Hopf linkoid and its states.

**Figure 3.**
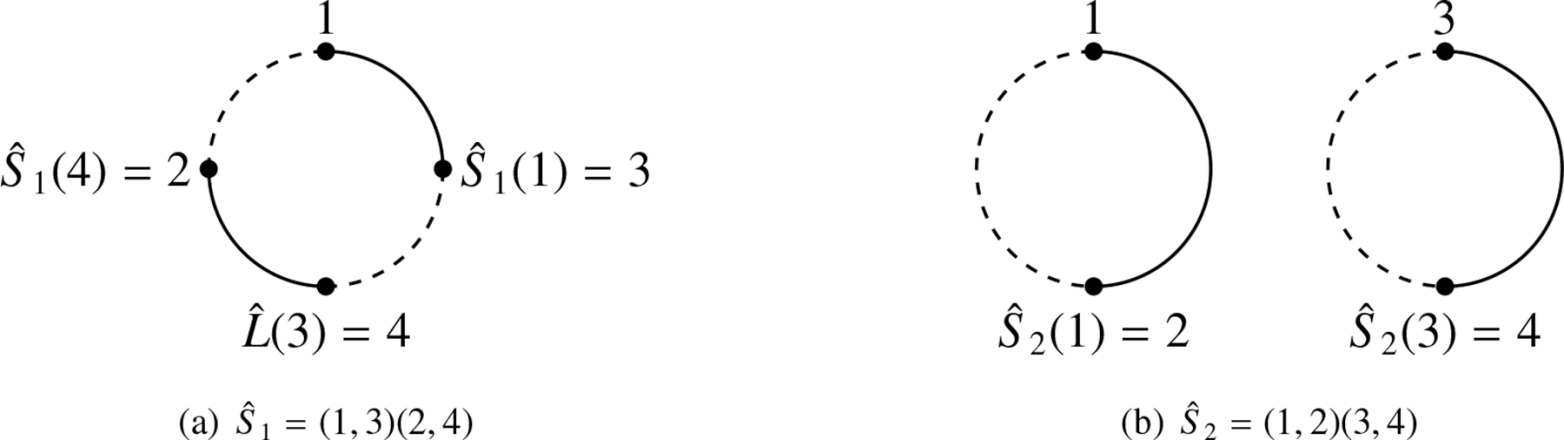
Segment cycles of two states.

**Example 1.** *Consider the linkoid diagram* L *as shown in* Figure 2(a). *The set* G *of all endpoints is* {1, 2, 3, 4}. *There are two states of* $L,S_{1}$, *and* $S_{2}$, *as shown in* Figures 2(b) *and* (c). *The associated pairings* ${\overset{ˆ}{S}}_{1}$ *and* ${\overset{ˆ}{S}}_{2}$ *are represented by the permutations* (1, 3) (2, 4) *and* (1, 2) (3, 4), *respectively. The segment cycles of states* $S_{1}$ *and* $S_{2}$ *are shown in* Figure 3. *Then*, ${\left|S_{1}\right|}_{\text{cyc}}=1$ *and* ${\left|S_{2}\right|}_{\text{cyc}}=2$.

### 2.2. Jones polynomial

The bracket polynomial of linkoids in $S^{2}$ or $R^{2}$ is defined through an extension of the bracket polynomial of links. The following initial conditions and diagrammatic relations are sufficient for the skein computation of the bracket polynomial of linkoids.

**Definition 2.1** *Let* L *be a linkoid diagram with* n *components. The bracket polynomial of the linkoid is uniquely determined by the following skein relation and initial conditions:*


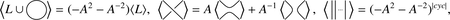


where |cyc| denotes the number of distinct segment cycles.

The bracket polynomial of L can be expressed as the following state sum expression:

$$\langle L\rangle =\underset{S}{\sum }A^{\sigma \left(S\right)}d^{{\left|S\right|}_{\text{circ}}-1}d^{{\left|S\right|}_{\text{cyc}}},$$

where S is a state corresponding to a choice of smoothing over all crossing points in L;σ(S) is the algebraic sum of the smoothing labels of $S;|S{|}_{\text{circ}}$ is the number of disjoint circles in $S,|S{|}_{\text{cyc}}$ is the number of distinct segment cycles in S; and $d=\left(-A^{2}-A^{-2}\right)$.

The *normalized bracket polynomial is defined* as follows:

$$f_{L}={\left(-A^{-3}\right)}^{-Wr(L)}\langle L\rangle ,$$

where Wr(L) is the writhe of the linkoid diagram L.

Now, consider curves in 3-space. A regular projection of curves fixed in 3-space can result in different linkoid diagrams depending on the projection direction chosen. Barkataki and Panagiotou in [25] define the bracket polynomial of curves in 3-space as the average of the bracket polynomial of a projection of the curve over all possible projection directions. This definition is made precise as follows:

**Definition 2.2.** *[*25, *Definition 4.1.] Let* L *be a collection of disjoint open or closed curves in the 3*-*space. Let* $(L{)}_{\xi }$ *denote the projection of* L *on a plane with normal vector* ξ. *The normalized bracket polynomial of* L *is defined as follows:*

$$f_{L}=\frac{1}{4\pi }\int_{\xi \in S^{2}}{\left(-A^{3}\right)}^{-Wr\left((L{)}_{\xi }\right)}\left\langle (L{)}_{\xi }\right\rangle dS,$$

where each $(L{)}_{\xi }$ is a linkoid diagram, and its bracket polynomial can be calculated using Definition 2.1. Note that the integral is taken over all vectors $\xi \in S^{2}$, excluding a set of measure zeros (corresponding to the irregular projections). This gives the Jones polynomial of a collection of disjoint open or closed curves in the 3-space with substitution $A=t^{-\frac{1}{4}}$.

**Proposition 2.1.** *[*25, *Proposition 4.1.]*

- For open curves, the Jones polynomial has real coefficients and is a continuous function of the curve coordinates.
- As the endpoints of the open curves tend to coincide in 3-space, the Jones polynomial tends to that of the corresponding link.

## 3. Localized Jones polynomials

The Jones polynomial of a collection of disjoint open or closed curves in 3-space describes the entanglement of the curves within the entire collection. However, in many applications, it is desirable to extract the local structural information of the curves. Two methods for localizing the Jones polynomial are proposed to capture the entanglement of a collection of curves and to meet the needs of practical applications.

### 3.1. Multi-scale Jones polynomial

Let L be a collection of disjoint open or closed curves in the 3-space. Given a segmentation $P_{n}=\left\{l_{1},l_{2},\ldots ,l_{n}\right\}$ of L, where each $l_{i}$ represents a finite curve segment of L, the segments $l_{i},1\leq i\leq n$ can be connected sequentially to reconstruct L.

To investigate the entanglement properties between each curve segment and its neighboring segments, multi-scale analysis is a suitable approach. Multi-scale analysis requires the definition of a distance metric between the curve segments. The distance between segments, denoted $d\left(l_{i},l_{j}\right)$, can be specified by different metrics, depending on the application context. In this study, for simplicity, we define the distance $d\left(l_{i},l_{j}\right)$ as the upper bound of the Eulerian distances between a point of one curve segment and another curve segment.

For any curve segment $l_{i}$, consider the set of segments within $P_{n}$ whose distances from $l_{i}$ fall within the range [r,R), where $r⩽d\left(l_{i},l_{j}\right)<R$. This set, which includes $l_{i}$ itself, is denoted as

$$P_{r,R}^{i}=\left\{l_{j}\in P_{n}|r⩽d\left(l_{i},l_{j}\right)<R\right\}\cup \left\{l_{i}\right\}.$$

The Jones polynomial of the set of curve segments $P_{r,R}^{i}$, denoted by $JP_{r,R}^{i}$, quantifies the entanglement of the curve segment $l_{i}$ with other segments as r and R vary. By selecting two sets of distance parameters, $\left\{r_{1},r_{2},\ldots ,r_{m}\right\}$ and $\left\{R_{1},R_{2},\ldots ,R_{m}\right\}$, with $r_{i}<R_{i}$ for 1≤i≤m, we obtain a set of characteristic polynomials for $l_{i}$:

$$\left\{JP_{r_{1},R_{1}}^{i},JP_{r_{2},R_{2}}^{i},\ldots ,JP_{r_{m},R_{m}}^{i}\right\}.$$

Applying this procedure to all curve segments in $P_{n}$, we obtain an n×m matrix:

$$\left(\begin{array}{cccc} JP_{r_{1},R_{1}}^{1} & JP_{r_{2},R_{2}}^{1} & \cdots & JP_{r_{m},R_{m}}^{1}\\ JP_{r_{1},R_{1}}^{2} & JP_{r_{2},R_{2}}^{2} & \cdots & JP_{r_{m},R_{m}}^{2}\\ \vdots & \vdots & \ddots & \vdots \\ JP_{r_{1},R_{1}}^{n} & JP_{r_{2},R_{2}}^{n} & \cdots & JP_{r_{m},R_{m}}^{n} \end{array}\right),$$

capturing both local and global entanglement properties for the collection of disjoint open or closed curves L.

Each matrix entry is a polynomial. For practical applications, the Jones polynomial can be evaluated at a specific parametrization, such as t=10, resulting in an n×m characteristic matrix for the segmentation $P_{n}$ of L:

$$mJ\left(P_{n}\right)=\left(\begin{array}{cccc} JP_{r_{1},R_{1}}^{1}(10) & JP_{r_{2},R_{2}}^{1}(10) & \cdots & JP_{r_{m},R_{m}}^{1}(10)\\ JP_{r_{1},R_{1}}^{2}(10) & JP_{r_{2},R_{2}}^{2}(10) & \cdots & JP_{r_{m},R_{m}}^{2}(10)\\ \vdots & \vdots & \ddots & \vdots \\ JP_{r_{1},R_{1}}^{n}(10) & JP_{r_{2},R_{2}}^{n}(10) & \cdots & JP_{r_{m},R_{m}}^{n}(10) \end{array}\right),$$

where each entry is a real number.

**Remark 3.1.** *For specific application contexts involving research objects that can be represented as a collection of disjoint open or closed curves* L, *the choice of segmentation* $P_{n}=\left\{l_{1},l_{2},\ldots ,l_{n}\right\}$ *plays a critical role. The choice of an appropriate segmentation, tailored to the requirements of the application*, *allows for a more precise capture of the entanglement properties inherent to the research objects, and, thus, a more accurate reflection of their characteristics. Similarly, the choice of parameters $\left\{r_{1},r_{2},\ldots ,r_{m}\right\}$ and* $\left\{R_{1},R_{2},\ldots ,R_{m}\right\}$ *influences the robustness and precision of the final assessment of the entanglement features of the objects*.

### 3.2. Persistent Jones polynomial

To address different application scenarios and to better capture the information about entanglement of curves in the 3-space, a second localization of the Jones polynomial has been proposed. This adaptation of the Jones polynomial for a collection of disjoint open or closed curves, aimed at quantifying the complexity of entanglement, serves as an extension of the classical Jones polynomial [25].

Let L be a collection of disjoint open or closed curves with a segmentation denoted by $P_{n}=\left\{l_{1},l_{2},\ldots ,l_{n}\right\}$, where $l_{i}$ is a segment of L. The segments $l_{i},1⩽i⩽n$, can be connected sequentially to reconstruct L. To effectively represent these multiple segments, both the Čech complex and the Vietoris-Rips complex, constructed here from the distance matrix,

$$d\left(P_{n}\right)=\left(\begin{array}{cccc} 0 & d\left(l_{1},l_{2}\right) & \cdots & d\left(l_{1},l_{n}\right)\\ d\left(l_{2},l_{1}\right) & 0 & \cdots & d\left(l_{2},l_{n}\right)\\ \vdots & \vdots & \ddots & \vdots \\ d\left(l_{n},l_{1}\right) & d\left(l_{n},l_{2}\right) & \cdots & 0 \end{array}\right)$$

from $P_{n}=\left\{l_{1},l_{2},\ldots ,l_{n}\right\}$, are suitable methods. Given the similarity between the Čech complex and the Vietoris-Rips complex, we will focus on the Vietoris-Rips complex in the following discussion. Let r denote the variable parameter of the Vietoris-Rips complex.

**Definition 3.1.** *A critical value of the Vietoris-Rips complex is a real number* r *such that, for any sufficiently small* ε>0, *the map* $K_{r-\varepsilon }↪K_{r+\varepsilon }$ *is an inclusion but not an isomorphism, where* $K_{r}$ *denotes the complex at the Vietoris-Rips parameter* r.

**Remark 3.2.** *For a segmentation* $P_{n}$ *of* L *into finite curve segments, the Vietoris-Rips complex has a finite number of critical values*.

Let $r_{0}<r_{1}<r_{2}<\cdots <r_{m}$ represent the critical values of the Vietoris-Rips complex for a segmentation $P_{n}$ of L. This generates a sequence of complexes from $P_{n}$ that form a filtration $ℱ\left(P_{n}\right)$:

$$K_{r_{0}}⫋K_{r_{1}}⫋K_{r_{2}}⫋\cdots ⫋K_{r_{m}},$$

where the final complex $K_{r_{m}}$ is an (n-1)-simplex. For any x<y, let $V_{x}^{y}:K_{x}↪K_{y}$ denote the inclusion map.

**Lemma 3.1.** *[*36, *Critical Value Lemma] If a closed interval* [x,y] *does not contain a critical value of the Vietoris-Rips complex, then* $V_{x}^{y}:K_{x}\to K_{y}$ *is an isomorphism*.

Within the filtration $ℱ\left(P_{n}\right)$, consider a complex $K_{r}$. Each vertex $v_{a}\subset K_{r}$ corresponds to a segment $l_{a}$ of the curve segments in $P_{n}$. An edge $\left\{v_{a},v_{b}\right\}\subset K_{r}$ indicates that the distance between segments $l_{a}$ and $l_{b}$ is less than the Vietoris-Rips parameter r. A simplex $\Delta =\left\{v_{a},v_{b},\ldots ,v_{t}\right\}\subset K_{r}$ means that the pair-wise distances among the corresponding segments $\left\{l_{a},l_{b},\ldots ,l_{t}\right\}$, collectively denoted by $\Delta \left(P_{n}\right)$, are all less than r.

**Definition 3.2.** *Let* K *be a simplicial complex. The maximal faces of* K *with respect to inclusion are named the facets of* K. The simplicial complex K, *characterized by the facets* $F_{1},\ldots ,F_{q}$, *is denoted by*

$$K=\left\langle F_{1},\ldots ,F_{q}\right\rangle ,$$

and the set $\left\{F_{1},\ldots ,F_{q}\right\}$ is called the set of facets of K.

Each complex within the filtration $ℱ\left(P_{n}\right)$ can be described by its facets. Thus, the filtration $ℱ\left(P_{n}\right)$ can be expressed as:

$$\left\langle F_{1}^{r_{0}},\ldots ,F_{q_{0}}^{r_{0}}\right\rangle ⫋\left\langle F_{1}^{r_{1}},\ldots ,F_{q_{1}}^{r_{1}}\right\rangle ⫋\ldots ⫋\left\langle F_{1}^{r_{m}},\ldots ,F_{q_{m}}^{r_{m}}\right\rangle .$$

**Definition 3.3.** *The birth of a facet* F *in the filtration* $ℱ\left(P_{n}\right)$ *is the smallest index* $r_{b}$ *such that* F *appears as a facet in the complex* $K_{r_{b}}$ *but not in* $K_{r_{b}-\varepsilon }$ *for any sufficiently small* ε>0.

The *death* of a facet F in the filtration $ℱ\left(P_{n}\right)$ is the largest index $r_{d}$ such that F is a facet in $K_{r_{d}}$ but not in $K_{r_{d}+\varepsilon }$ for any sufficiently small ε>0.

The *life-span* of a facet F in the filtration $ℱ\left(P_{n}\right)$ is the interval $\left[r_{b},r_{d}\right]$.

Therefore, $ℱ\left(P_{n}\right)$ can be represented as a sequence of facets, each associated with a birth-and-death interval,

$$\left\{\left(F_{1},\left[r_{b}^{1},r_{d}^{1}\right]\right),\left(F_{2},\left[r_{b}^{2},r_{d}^{2}\right]\right),\ldots ,\left(F_{t},\left[r_{b}^{t},r_{d}^{t}\right]\right)\right\}.$$

Similar to persistent barcodes in persistent homology, a barcode can represent the facets within a filtration. For any given dimension, each bar corresponds to a facet, with the start and end points of the bar indicating the birth and death of the associated facet, respectively.

**Definition 3.4.** *The barcode* $B\left(P_{n}\right)$ *for the filtration of facets* $ℱ\left(P_{n}\right)$ *consists of horizontal line segments* $\left[r_{i},r_{j}\right]$, *where* $r_{i}\leq r_{j}$, *representing the birth and death times of the associated facet*.

A barcode provides a visual representation of a filtration as a collection of horizontal line segments on a plane, where the horizontal axis corresponds to the parameter, and the vertical axis represents an ordering of the facets.

**Definition 3.5.** *For each facet* $F=\left\{v_{a},v_{b},\ldots ,v_{t}\right\}$ *in the filtration* $ℱ\left(P_{n}\right)$, *there exists a corresponding subset of segments* $F\left(P_{n}\right)=\left\{l_{a},l_{b},\ldots ,l_{t}\right\}$ *in the segmentation* $P_{n}$. *The Jones polynomial of* $F\left(P_{n}\right)$, *denoted by* $JF\left(P_{n}\right)$, *is defined as the weight of the facet* F. *Consequently, the Jones polynomial can be treated as a weighting function for filtration* $ℱ\left(P_{n}\right)$. *The resulting weighted filtration is denoted by* $Jℱ\left(P_{n}\right)$,

$$\left\{\left(F_{1},\left[r_{b}^{1},r_{d}^{1}\right],JF_{1}\left(P_{n}\right)\right),\left(F_{2},\left[r_{b}^{2},r_{d}^{2}\right],JF_{2}\left(P_{n}\right)\right),\ldots ,\left(F_{t},\left[r_{b}^{t},r_{d}^{t}\right],JF_{t}\left(P_{n}\right)\right)\right\}$$

referred to as the persistent Jones polynomial of the segmentation $P_{n}$ of L.

Since filtration $ℱ\left(P_{n}\right)$ can be expressed by a barcode of facets, the persistent Jones polynomial of the segmentation $P_{n}$ of L can also be expressed by a barcode, with the Jones polynomials of the associated facets as weights. The weights in the persistent Jones polynomial of $P_{n}$ are polynomials. To improve applicability in specific scenarios, setting the Jones polynomial variable t=10 converts these weights to real numbers, producing a real-number weighted barcode $BJ\left(P_{n}\right)(10)$.

**Remark 3.3.** *The persistent Jones polynomial and the classical persistent homology perform a multi-scale analysis of the data through filtration using Vietoris-Rips complexes or other types of complexes. However, they focus on different aspects of data analysis. The persistent Jones polynomial uses tools from geometric topology to capture the entanglement complexity of curves in 3-space, whereas classical persistent homology uses methods from algebraic topology to investigate the behavior of connected components, one-dimensional loops, two-dimensional cavities, and high-dimensional cavities (i.e., generators of homology groups) within a point cloud data*.

## 4. Stability

The stability of a model is characterized by the property that small perturbations in the collection of disjoint open or closed curves L result in only minor variations in the localized measures of the multi-scale Jones polynomial and the persistent Jones polynomial.

Let f:L→f(L) be a continuous function acting on a collection of disjoint open or closed curves L in the 3-space. The difference between f(L) and L is measured using the supremum norm $\|f(L)-L{\|}_{\infty }$, defined as:

$$\|f(L)-L{\|}_{\infty }=\underset{x\in L}{\text{sup}}|f(x)-x|.$$

For a given segmentation $P_{n}=\left\{l_{1},l_{2},\ldots ,l_{n}\right\}$ of L, where each $l_{i}(1⩽i⩽n)$ is a finite curve segment and L can be reconstructed by connecting these segments end-to-end, consider a continuous mapping f:L→f(L) such that $\|f(L)-L{\|}_{\infty }<\varepsilon$ for a sufficiently small ε>0. This induces a corresponding segmentation $f\left(P_{n}\right)=\left\{f\left(l_{1}\right),f\left(l_{2}\right),\ldots ,f\left(l_{n}\right)\right\}$ of f(L).

The characteristic matrices of the multi-scale Jones polynomial for $P_{n}$ and $f\left(P_{n}\right)$, denoted $mJ\left(P_{n}\right)$ and $mJ\left(f\left(P_{n}\right)\right)$, respectively, exhibit only minor differences at the corresponding positions. Furthermore, the weighted Bottleneck distance between the weighted persistence diagrams of persistent Jones polynomials for $P_{n}$ and $f\left(P_{n}\right)$ is also minimal.

**Remark 4.1.** *The Jones polynomial evaluated at* t=10 *can be viewed as a function on collections of curves in 3-space. Let* L *be a collection of curves in* $R^{3}$. *According to Proposition 2.1, let* f:L→f(L) *be a continuous function. If* $\|f(L)-L{\|}_{\infty }<\varepsilon$ *for all sufficiently small* ε>0, *then $|J(L)(10)-J(f(L))(10)|<\varepsilon_{J}$ for some sufficiently small $\varepsilon_{J}>0$*.

### 4.1. Stability of multi-scale Jones polynomial

Let L be a collection of disjoint open or closed curves in the 3-space, and let $P_{n}=\left\{l_{1},l_{2},\ldots ,l_{n}\right\}$ denote a segmentation of L into n segments. Consider a continuous function f:L→f(L), which induces a corresponding segmentation of f(L), represented by $f\left(P_{n}\right)=\left\{f\left(l_{1}\right),f\left(l_{2}\right),\ldots ,f\left(l_{n}\right)\right\}$.

**Proposition 4.1.** *Suppose* f:L→f(L) *is a continuous function such that* $\|f(L)-L{\|}_{\infty }<\varepsilon$ *for all sufficiently small* ε>0. *Then, the two sets of curve segments* $f\left(P_{r,R}^{i}\right)$ and $f(P{)}_{r,R}^{i}$ *are equal*:

$$f\left(P_{r,R}^{i}\right)=f(P{)}_{r,R}^{i},$$

where:

- $f\left(P_{r,R}^{i}\right)$ is the image of the set $P_{r,R}^{i}$ under the function f:L→f(L);
- $f(P{)}_{r,R}^{i}$ represents the set of curve segments in the segmentation $f\left(P_{n}\right)$ such that their distance from $f\left(l_{i}\right)$ is within [r,R), including of $f\left(l_{i}\right)$ itself.

*Proof*. By definition, we have

$$P_{r,R}^{i}=\left\{l_{j}\in P_{n}|r\leq d\left(l_{i},l_{j}\right)<R\right\}\cup \left\{l_{i}\right\},$$

$$f\left(P_{r,R}^{i}\right)=\left\{f\left(l_{j}\right)\in P_{n}|r\leq d\left(l_{i},l_{j}\right)<R\right\}\cup \left\{f\left(l_{i}\right)\right\},$$

and

$$f(P{)}_{r,R}^{i}=\left\{f\left(l_{j}\right)\in f\left(P_{n}\right)|r\leq d\left(f\left(l_{i}\right),f\left(l_{j}\right)\right)<R\right\}\cup \left\{f\left(l_{i}\right)\right\},$$

for any $l_{k}\in P_{r,R}^{i}$, and it holds that $r\leq d\left(l_{i},l_{k}\right)<R$. Since $\|f(L)-L{\|}_{\infty }<\varepsilon$, the difference between each segment of the curve and its image under f is less than $\varepsilon :{\left\|f\left(l_{i}\right)-l_{i}\right\|}_{\infty }<\varepsilon$ and ${\left\|f\left(l_{k}\right)-l_{k}\right\|}_{\infty }<\varepsilon$, and, thus, the distances satisfy $d\left(f\left(l_{i}\right),l_{i}\right)<\varepsilon$ and $d\left(f\left(l_{k}\right),l_{k}\right)<\varepsilon$.


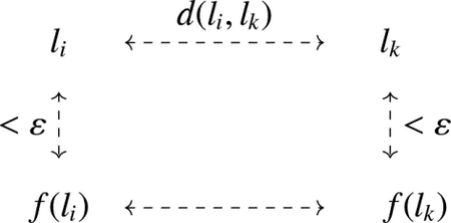


Then, we have:

$$d\left(l_{i},l_{k}\right)-2\varepsilon <d\left(f\left(l_{i}\right),f\left(l_{k}\right)\right)<d\left(l_{i},l_{k}\right)+2\varepsilon .$$

Therefore,

$$r-2\varepsilon <d\left(f\left(l_{i}\right),f\left(l_{k}\right)\right)<R+2\varepsilon ,$$

which implies $f\left(l_{k}\right)\in f(P{)}_{r-2\varepsilon ,R+2\varepsilon }^{i}$. Thus, $f\left(P_{r,R}^{i}\right)\subseteq f(P{)}_{r-2\varepsilon ,R+2\varepsilon }^{i}$.

Similarly, we can show that:

$$f(P{)}_{r-2\varepsilon ,R+2\varepsilon }^{i}\subseteq f\left(P_{r-4\varepsilon ,R+4\varepsilon }^{i}\right).$$

Consequently, we obtain:

$$f\left(P_{r,R}^{i}\right)\subseteq f(P{)}_{r-2\varepsilon ,R+2\varepsilon }^{i}\subseteq f\left(P_{r-4\varepsilon ,R+4\varepsilon }^{i}\right).$$

Since ε>0 is sufficiently small, we conclude that $P_{r-4\varepsilon ,R+4\varepsilon }^{i}=P_{r,R}^{i},f(P{)}_{r-2\varepsilon ,R+2\varepsilon }^{i}=f(P{)}_{r,R}^{i}$, and $f\left(P_{r,R}^{i}\right)=f(P{)}_{r,R}^{i}$. □

**Theorem 4.1.** *Suppose* f:L→f(L) *is a continuous function such that* $\|f(L)-L{\|}_{\infty }<\varepsilon$ *for all sufficiently small* ε>0. *Consider two sets of distances* $\left\{r_{1},r_{2},\ldots ,r_{m}\right\}$ *and* $\left\{R_{1},R_{2},\ldots ,R_{m}\right\}$. *There exist two characteristic matrices for the segmentation* $P_{n}$ *of* L *and for the segmentation* $f\left(P_{n}\right)$ *of* f(L), *given by*

$$mJ\left(P_{n}\right)=\left(\begin{array}{cccc} JP_{r_{1},R_{1}}^{1}(10) & JP_{r_{2},R_{2}}^{1}(10) & \cdots & JP_{r_{m},R_{m}}^{1}(10)\\ JP_{r_{1},R_{1}}^{2}(10) & JP_{r_{2},R_{2}}^{2}(10) & \cdots & JP_{r_{m},R_{m}}^{2}(10)\\ \vdots & \vdots & \ddots & \vdots \\ JP_{r_{1},R_{1}}^{n}(10) & JP_{r_{2},R_{2}}^{n}(10) & \cdots & JP_{r_{m},R_{m}}^{n}(10) \end{array}\right),$$

$$mJ\left(f\left(P_{n}\right)\right)=\left(\begin{array}{cccc} Jf(P{)}_{r_{1},R_{1}}^{1}(10) & Jf(P{)}_{r_{2},R_{2}}^{1}(10) & \cdots & Jf(P{)}_{r_{m},R_{m}}^{1}(10)\\ Jf(P{)}_{r_{1},R_{1}}^{2}(10) & Jf(P{)}_{r_{2},R_{2}}^{2}(10) & \cdots & Jf(P{)}_{r_{m},R_{m}}^{2}(10)\\ \vdots & \vdots & \ddots & \vdots \\ Jf(P{)}_{r_{1},R_{1}}^{n}(10) & Jf(P{)}_{r_{2},R_{2}}^{n}(10) & \cdots & Jf(P{)}_{r_{m},R_{m}}^{n}(10) \end{array}\right).$$

Then, the difference between the corresponding entries in these two matrices is less than $\varepsilon_{J}$, where $\varepsilon_{J}$ is sufficiently small.

*Proof*. We have

$${\left\|P_{r_{j},R_{j}}^{i}-f\left(P_{r_{j},R_{j}}^{i}\right)\right\|}_{\infty }\leq \|L-f(L){\|}_{\infty }<\varepsilon .$$

From Remark 4.1, it follows that

$$\left|JP_{r_{j},R_{j}}^{i}(10)-Jf\left(P_{r_{j},R_{j}}^{i}\right)(10)\right|<\varepsilon_{J},$$

where $\varepsilon_{J}$ is sufficiently small. By Proposition 4.1, we know that $f\left(P_{r,R}^{i}\right)=f(P{)}_{r,R}^{i}$. Therefore,

$$\left|JP_{r_{j},R_{j}}^{i}(10)-Jf(P{)}_{r_{j},R_{j}}^{i}(10)\right|<\varepsilon_{J},$$

where $\varepsilon_{J}$ is sufficiently small. □

**Remark 4.2.** *The stability of the method used in* [20] *can be proved in a manner similar to that of Theorem 4.1. Suppose* f:L→f(L) *is a continuous function such that* $\|f(L)-L{\|}_{\infty }<\varepsilon$ *for all sufficiently small* ε>0.

For the Gauss linking integral, there exist two Gauss linking integral matrices for the segmentation $P_{n}=\left\{l_{1},l_{2},\ldots ,l_{n}\right\}$ of a collection of disjoint open or closed curves L in 3-space, as well as for $f\left(P_{n}\right)$ of f(L). These matrices are given by:

$$GL\left(P_{n}\right)=\left(\begin{array}{cccc} g\left(l_{1},l_{1}\right) & g\left(l_{1},l_{2}\right) & \cdots & g\left(l_{1},l_{n}\right)\\ g\left(l_{2},l_{1}\right) & g\left(l_{2},l_{2}\right) & \cdots & g\left(l_{2},l_{n}\right)\\ \vdots & \vdots & \ddots & \vdots \\ g\left(l_{n},l_{1}\right) & g\left(l_{n},l_{2}\right) & \cdots & g\left(l_{n},l_{n}\right) \end{array}\right),$$

$$GL\left(f\left(P_{n}\right)\right)=\left(\begin{array}{cccc} g\left(f\left(l_{1}\right),f\left(l_{1}\right)\right) & g\left(f\left(l_{1}\right),f\left(l_{2}\right)\right) & \cdots & g\left(f\left(l_{1}\right),f\left(l_{n}\right)\right)\\ g\left(f\left(l_{2}\right),f\left(l_{1}\right)\right) & g\left(f\left(l_{2}\right),f\left(l_{2}\right)\right) & \cdots & g\left(f\left(l_{2}\right),f\left(l_{n}\right)\right)\\ \vdots & \vdots & \ddots & \vdots \\ g\left(f\left(l_{n}\right),f\left(l_{1}\right)\right) & g\left(f\left(l_{n}\right),f\left(l_{2}\right)\right) & \cdots & g\left(f\left(l_{n}\right),f\left(l_{n}\right)\right) \end{array}\right),$$

where

$$g\left(l_{i},l_{j}\right)=\left\{\begin{array}{ll} GL\left(l_{i},l_{j}\right), & \text{if}\;l_{i}\cap l_{j}=\emptyset ;\\ 0, & \text{otherwise}. \end{array}\right.$$

Here, $GL\left(l_{i},l_{j}\right)$ denotes the Gauss linking integral of the curve segments $l_{i}$ and $l_{j}$.

As stated in [24, page 3], we can treat the Gauss linking integral of a curve as a continuous function of the coordinates of the curve. Similar to Theorem 4.1, we also have

$$\left|g\left(l_{i},l_{j}\right)-g\left(f\left(l_{i}\right),f\left(l_{j}\right)\right)\right|<\varepsilon_{GL}$$

for sufficiently small $\varepsilon_{GL}$.

### 4.2. Stability of the persistence Jones polynomial

In this section, we state and prove the stability of the persistent Jones polynomial, which asserts that small changes in the collection of disjoint open or closed curves L lead to only small changes in the persistent Jones polynomial. As discussed in Section 3.2, the persistent Jones polynomial is represented by a weighted Jones polynomial filtration, which can be expressed as a Jones polynomial weight barcode of facets. For a given barcode, there is a corresponding diagram where each bar in the barcode can be mapped to a point in the diagram. The x-coordinate of this point represents the birth time of the corresponding bar, while the y-coordinate represents its death time.

Let L be a collection of disjoint open or closed curves in the 3-space, and let $P_{n}=\left\{l_{1},l_{2},\ldots ,l_{n}\right\}$ denote a segmentation of L into n segments. Consider a continuous function f:L→f(L), which induces a corresponding segmentation of f(L), represented by $f\left(P_{n}\right)=\left\{f\left(l_{1}\right),f\left(l_{2}\right),\ldots ,f\left(l_{n}\right)\right\}$. Let $r_{1}<r_{2}<\cdots <r_{m}$ be the critical values of the Vietoris-Rips complex of $P_{n}$. We denote an interleaved sequence ${\left(b_{i}\right)}_{i=0,1,\ldots ,m}$ such that $b_{i-1}<r_{i}<b_{i}$ for all i. We set $b_{-1}=r_{0}=-\infty$ and $b_{m+1}=r_{m+1}=+\infty$.

**Figure 4.**
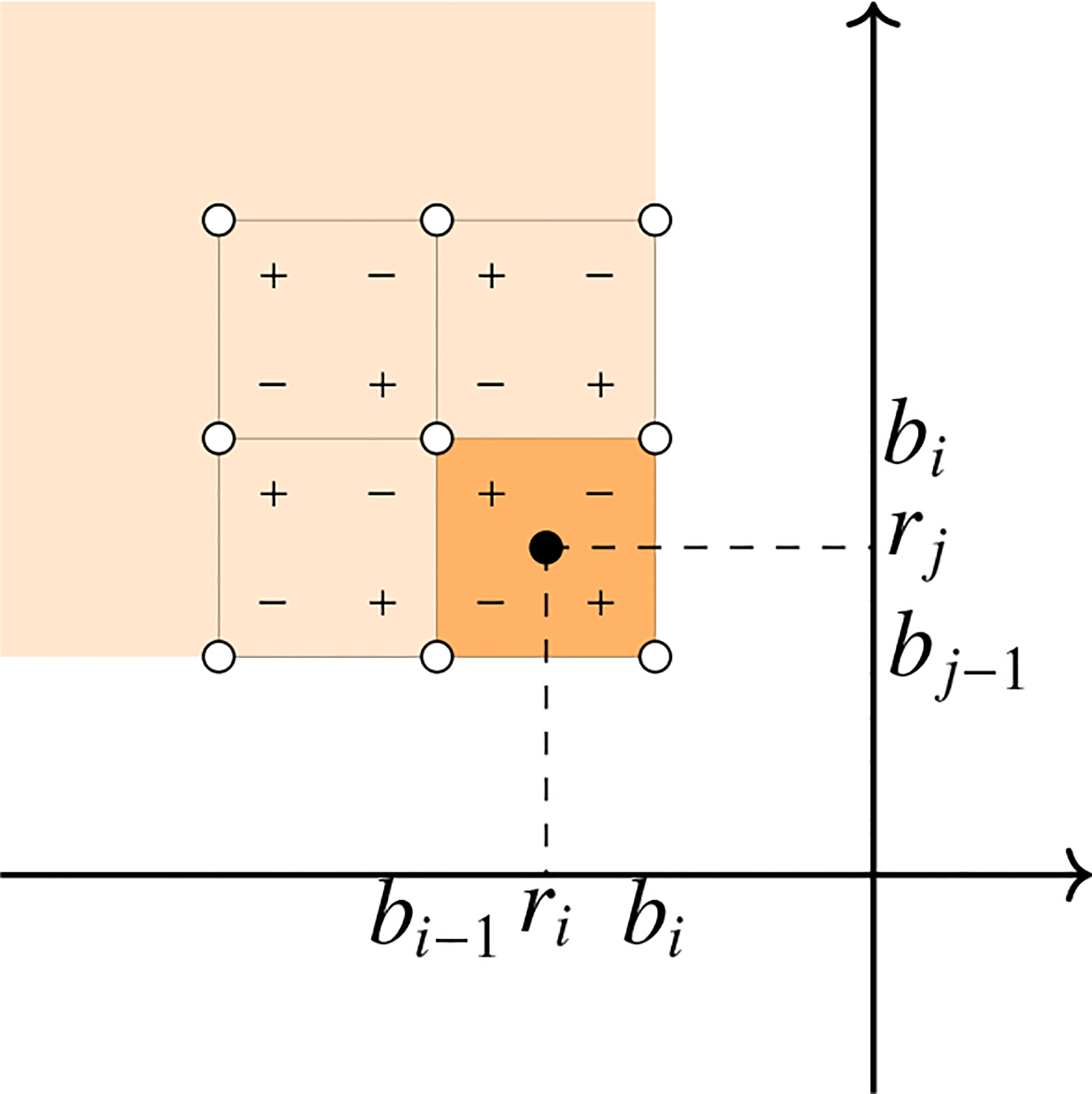
The multiplicity of the point $\left(r_{i},r_{j}\right)$ is the alternating sum at the corners of the lower right square. When other multiplicities are added, cancellations between plus and minus signs occur.

For two integers 0⩽i<j⩽m+1 and a fixed integer k, we define the *multiplicity* of the pair ($r_{i},r_{j}$) as

$$\mu_{i}^{j}=\beta_{b_{i-1}}^{b_{j}}-\beta_{b_{i}}^{b_{j}}+\beta_{b_{i}}^{b_{j-1}}-\beta_{b_{i-1}}^{b_{j-1}},$$

where $\beta_{x}^{y}$ is the number of k-facets contained in $K_{x}$ that remain in $K_{y}$ for all -∞⩽x⩽y⩽+∞. To visualize this definition, consider $\beta_{x}^{y}$ as the value of a function β at the point $\left(r_{i},r_{j}\right)\in {\bar{R}}^{2}$, where $\bar{R}=R\cup \{-\infty ,+\infty \}$. Thus, $\mu_{i}^{j}$ is the alternating sum of β in the corners of the box $\left[b_{i-1},b_{i}\right]\times \left[b_{j-1},b_{j}\right]$, as depicted in Figure 4.

Note that if x and $x^{\prime }$ are in the open interval $\left(r_{i},r_{i+1}\right)$, and y and $y^{\prime }$ are in $\left(r_{j-1},r_{j}\right)$, then $\beta_{x}^{y}=\beta_{x^{\prime }}^{y^{\prime }}$. Therefore, the multiplicities $\mu_{i}^{j}$ are well-defined and always nonnegative.

**Definition 4.1.** *The diagram* $D\left(P_{n}\right)\subset {\bar{R}}^{2}$ *of the persistent Jones polynomial for* $P_{n}$ *consists of points* $\left(r_{i},r_{j}\right)$ *with Jones polynomial weights, counted with multiplicity* $\mu_{i}^{j}$ *for 0≤i<j≤m+1*, *along with all points on the diagonal, which are counted with infinite multiplicity*.

Each off-diagonal point in the diagram represents the lifespan of a k-facet in the filtration. Similar to the weighted persistent barcode, the Jones polynomial corresponding to the set of curve segments for the k-facet can be used as the weight. This approach results in a weighted persistent diagram for the persistent Jones polynomial.

#### 4.2.1. Bottleneck distance

The Bottleneck distance is a classical measure used to quantify the difference between two persistent diagrams. It naturally extends to the comparison of weighted persistent diagrams, allowing for a precise demonstration of the variations between two persistent Jones polynomials.

To better capture the differences between persistent Jones polynomials, we apply a slight modification to the traditional definition of the Bottleneck distance. Let C and 𝒟 be two multi-sets of pairs (⟨a,b⟩,w), where ⟨a,b⟩ denotes an interval that can be any well-defined member of the set {[a,b],[a,b),(a,b],(a,b)}, and w∈R represents the weight of the interval ⟨a,b⟩.

A *matching* between the sets 𝒞 and 𝒟 is defined as a collection of pairs χ={(I,J)∈C×𝒟}, where each element I∈C and each element J∈𝒟 appears in at most one pair within χ. A matching forms a bijection between a subset of C and a subset of 𝒟. If a pair (I,J)∈χ, we say that I is *matched* with J. Conversely, if an element I does not appear in any pair, it is considered *unmatched*.

The *cost c(I,J)* of the matching elements $I=\left(\langle a,b\rangle ,w_{1}\right)$ and $J=\left(\langle c,d\rangle ,w_{2}\right)$ is defined as follows:

$$c(I,J)=\text{max}\left\{|c-a|,|d-b|,\left|w_{1}-w_{2}\right|\right\}.$$

Similarly, the cost c(I) of leaving an unmatched element I is defined as:

$$c(I)=\frac{b-a}{2}.$$

Finally, the cost of a matching χ is given by:

$$c(\chi )=\text{max}\left(\underset{(I,J)\in \chi }{\text{sup}}c(I,J),\underset{\text{unmatched}\;I\in 𝒞\cup 𝒟}{\text{sup}}c(I)\right).$$

**Definition 4.2.** *The weighted Bottleneck distance between* C *and* 𝒟 *is defined as*

$$d_{B}(C,𝒟)=\text{inf}\{c(\chi )|\chi \;\text{is}\;\text{a}\;\text{matching}\;\text{between}\;𝒞\;\text{and}\;𝒟\}.$$

The modified Bottleneck distance increases the emphasis on weight factors compared to the classical Bottleneck distance.

#### 4.2.2. Stability

Let L be a collection of disjoint open or closed curves in the 3-space, and let $P_{n}=\left\{l_{1},l_{2},\ldots ,l_{n}\right\}$ denote a segmentation of L into n segments. Consider a continuous function f:L→f(L), which induces a segmentation of f(L), denoted by $f\left(P_{n}\right)=\left\{f\left(l_{1}\right),f\left(l_{2}\right),\ldots ,f\left(l_{n}\right)\right\}$.

There are two persistent Jones polynomials based on $P_{n}$ and $f\left(P_{n}\right)$, represented as $Jℱ\left(P_{n}\right)$ and $Jℱ\left(f\left(P_{n}\right)\right)$, respectively. The facet weights in these persistent Jones polynomials are polynomials. By setting the Jones polynomial variable t=10, the weight of each facet is converted to a real number. Thus, the converted persistent Jones polynomials can be denoted by $Jℱ\left(P_{n}\right)(10)$ and $Jℱ\left(f\left(P_{n}\right)\right)(10)$. These can be expressed using weighted persistent diagrams, denoted by $D\left(P_{n}\right)$ and $D\left(f\left(P_{n}\right)\right)$.

Suppose ‖f(L)-L‖<ε for all sufficiently small ε>0. Then, for all $l_{p_{i}}\in P_{n}(p)$, we have ${\left\|l_{p_{i}}-f\left(l_{p_{i}}\right)\right\|}_{\infty }<\varepsilon$. Let $l_{p_{i}},l_{p_{j}}$ be any two curve segments in $P_{n}(p)$. Then, there exists


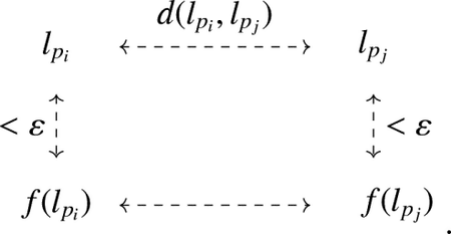


Thus,

$$d\left(l_{p_{i}},l_{p_{j}}\right)-2\varepsilon \leq d\left(f\left(l_{p_{i}}\right),f\left(l_{p_{j}}\right)\right)\leq d\left(l_{p_{i}},l_{p_{j}}\right)+2\varepsilon .$$

There is an important lemma, proved in [36].

**Lemma 4.1.** *[*36, *Box Lemma] For* a<b<c<d, *let* R=[a,b]×[c,d] *be a box in* $R^{2}$, *and let* $R_{2\varepsilon }=[a+2\varepsilon ,b-2\varepsilon ]\times [c+2\varepsilon ,d-2\varepsilon ]$ *be the box obtained by shrinking* R *on all sides by* 2ε. *It follows that*:

$$\#\left(D\left(P_{n}\right)\cap R_{2\varepsilon }\right)⩽\#\left(D\left(f\left(P_{n}\right)\right)\cap R\right).$$

**Theorem 4.2.** *Let* L *be a collection of disjoint open or closed curves, and let* $P_{n}=\left\{l_{1},l_{2},\ldots ,l_{n}\right\}$ *denote a segmentation of* L *into* n *segments. Suppose* f:L→f(L) *is a continuous function such that* $\|f(L)-L{\|}_{\infty }<\varepsilon$ *for all sufficiently small* ε>0. *Then, the weighted Bottleneck distance between the weighted persistent diagrams of the persistent Jones polynomials*, $d_{B}\left(D\left(P_{n}\right),D\left(f\left(P_{n}\right)\right)\right)$, *is sufficiently small*.

*Proof*. Let L be a collection of disjoint open or closed curves in 3-space, and let $P_{n}=\left\{l_{1},l_{2},\ldots ,l_{n}\right\}$ denote a segmentation of L into n segments. Consider a continuous function f:L→f(L), which induces a segmentation of f(L), denoted by $f\left(P_{n}\right)=\left\{f\left(l_{1}\right),f\left(l_{2}\right),\ldots ,f\left(l_{n}\right)\right\}$. There are two persistent Jones polynomial diagrams for $P_{n}$ and $f\left(P_{n}\right)$, denoted by $D\left(P_{n}\right)$ and $D\left(f\left(P_{n}\right)\right)$.

Consider the minimum distance between two distinct off-diagonal points or between an off-diagonal point and the diagonal:

$$\delta_{L}=\text{min}\left\{\|p-q{\|}_{\infty }|p\neq q\in D\left(P_{n}\right)-\Delta \right\}.$$

Assuming that ε>0 is sufficiently small, we take $\varepsilon <\delta_{L}/4$.

**Figure 5.**
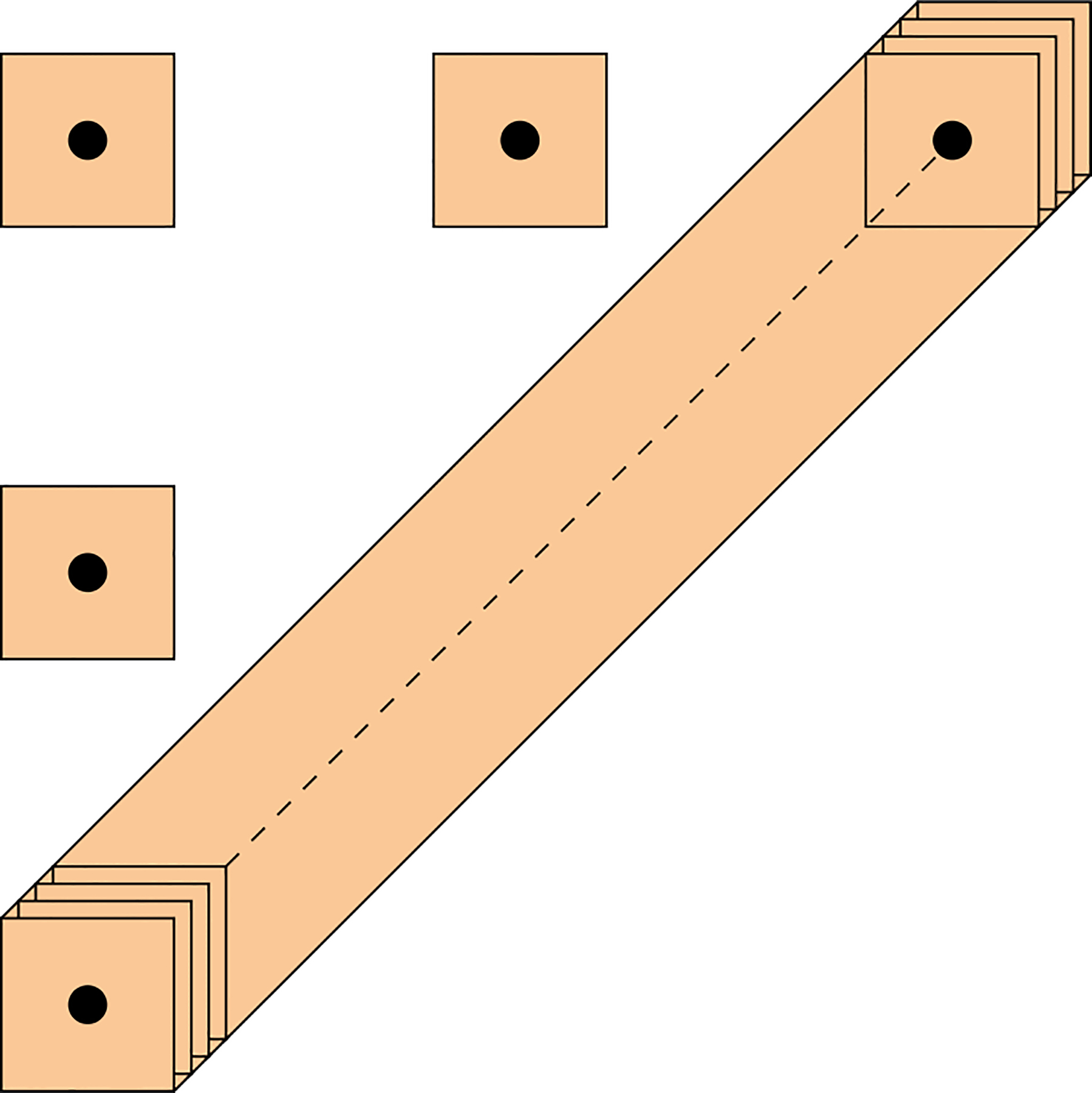
The shaded squares are centered at the black points of $D\left(P_{n}\right)$.

By drawing cubes of radius 2ε around points in $D\left(P_{n}\right)$, we obtain a thickened diagonal plane along with a finite set of disjoint cubes that are also disjoint from the thickened diagonal, as shown in Figure 5.

Let μ denote the multiplicity of a point p in $D\left(P_{n}\right)\setminus \Delta$, and let ${□}_{2\varepsilon }$ represent the cube centered at p with radius 2ε. According to Lemma 4.1,

$$\mu \leq \#\left(D\left(f\left(P_{n}\right)\right)\cap {□}_{2\varepsilon }\right)\leq \#\left(D\left(P_{n}\right)\cap {□}_{4\varepsilon }\right).$$

Since $4\varepsilon <\delta_{L},p$ is the only point in $D\left(P_{n}\right)$ within ${□}_{4\varepsilon }$, which implies that $\#\left(D\left(f\left(P_{n}\right)\right)\cap {□}_{2\varepsilon }\right)=\mu$.

Now, let p=(x,y) be an off-diagonal point in the diagram $D\left(P_{n}\right)$ with multiplicity μ=1. A corresponding collection of curve segments $P_{n}(p)=\left\{l_{p_{1}},l_{p_{2}},\ldots ,l_{p_{t}}\right\}$ exists in $P_{n}$. Applying the continuous function f to these segments yields the transformed collection $f\left(P_{n}(p)\right)=\left\{f\left(l_{p_{1}}\right),f\left(l_{p_{2}}\right),\ldots ,f\left(l_{p_{t}}\right)\right\}$.

According to the definition of the persistent Jones polynomial, the point p=(x,y) indicates that $P_{n}(p)=\left\{l_{p_{1}},l_{p_{2}},\ldots ,l_{p_{1}}\right\}$ forms a facet with birth x and death y in the filtration $ℱ\left(P_{n}\right)$. This implies that any two segments in $P_{n}(p)$ are at a distance less than x, and any segment $l\in P_{n}\setminus P_{n}(p)$ is at least a distance y from all $l_{p_{i}}\in P_{n}(p)$, for 1≤i≤t.

Since

$$d\left(l_{p_{i}},l_{p_{j}}\right)-2\varepsilon \leq d\left(f\left(l_{p_{i}}\right),f\left(l_{p_{j}}\right)\right)\leq d\left(l_{p_{i}},l_{p_{j}}\right)+2\varepsilon ,$$

then any two curve segments in $f\left(P_{n}(p)\right)$ are within a distance of x+2ε. Furthermore, for $f(l)\in f\left(P_{n}\setminus P_{n}(p)\right)$ and $f\left(l_{p_{i}}\right)\in f\left(P_{n}(p)\right)$, the distance is at least y-2ε.

Thus, the segment collection $f\left(P_{n}(p)\right)$ forms a facet within $ℱ\left(f\left(P_{n}\right)\right)$, corresponding to a point f(p) in $D\left(f\left(P_{n}\right)\right)$, within the region [x,x+2ε]×[y-2ε,y], indicating that $\|f(p)-p{\|}_{\infty }<2\varepsilon$. Moreover, we have $\#\left(D\left(f\left(P_{n}\right)\right)\cap {□}_{2\varepsilon }\right)=1$; hence, f(p) is the only point in $D\left(f\left(P_{n}\right)\right)\cap {□}_{2\varepsilon }$.

For off-diagonal points $p^{1}=p^{2}=\cdots =p^{\mu }=(x,y)$ with μ>1, this indicates μ distinct facets in $ℱ\left(P_{n}\right)$ with the same birth x and death y, but distinct curve segment collections $P_{n}\left(p^{1}\right),\;P_{n}\left(p^{2}\right),\ldots ,P_{n}\left(p^{\mu }\right)$. Analogous to the case when μ=1, the images $f\left(p^{1}\right),f\left(p^{2}\right),\ldots ,f\left(p^{\mu }\right)$ lie within [x,x+2ε]×[y-2ε,y], which gives ${\left\|f\left(p^{i}\right)-p^{i}\right\|}_{\infty }<2\varepsilon$ for 1≤i≤μ. Moreover, we have that $\#\left(D\left(f\left(P_{n}\right)\right)\cap {□}_{2\varepsilon }\right)=\mu$; hence, $f\left(p^{1}\right),f\left(p^{2}\right),\ldots ,f\left(p^{\mu }\right)$ are the μ points in $D\left(f\left(P_{n}\right)\right)\cap {□}_{2\varepsilon }$.

The weights of p and f(p) are $JP_{n}(p)(10)$ and $Jf\left(P_{n}(p)\right)(10)$, respectively. Since

$${\left\|f\left(P_{n}(p)\right)-P_{n}(p)\right\|}_{\infty }\leq \|f(L)-L{\|}_{\infty }<\varepsilon ,$$

by Remark 4.1, the weight difference satisfies $\left|Jf\left(P_{n}(p)\right)(10)-JP_{n}(p)(10)\right|<\varepsilon_{J}$, where $\varepsilon_{J}>0$ is sufficiently small.

After examining all the off-diagonal points in $D\left(P_{n}\right)$, the only points in $D\left(f\left(P_{n}\right)\right)$ that are not images f(p) for some $p\in \left(D\left(P_{n}\right)\setminus \Delta \right)$ lie beyond 2ε from $D\left(P_{n}\right)\setminus \Delta$.

Let $q\in D\left(f\left(P_{n}\right)\right)$ be a point for which there is no corresponding point $p\in D\left(P_{n}\right)$ such that f(p)=q. Suppose the distance from q to Δ is greater than 2ε. Then, there exists a square ${□}_{2\varepsilon }^{q}$ centered at q with a radius of 2ε such that $D\left(P_{n}\right)\cap {□}_{2\varepsilon }^{q}=\emptyset$. This contradicts Lemma 4.1, which states that $1\leq \#\left(D\left(P_{n}\right)\cap {□}_{2\varepsilon }^{q}\right)\neq 0$. Therefore, the distance from q to Δ must be less than 2ε.

There exists a natural matching between $D\left(P_{n}\right)$ and $D\left(f\left(P_{n}\right)\right)$, represented as $\chi =\{(p,f(p))|p\in \left.D\left(P_{n}\right)\setminus \Delta \right\}$, with unmatched points in $D\left(f\left(P_{n}\right)\right)$ regarded as not corresponding to any point in $D\left(P_{n}\right)$. Therefore, by the definition of the weighted Bottleneck distance,

$$d_{B}\left(D\left(P_{n}\right),D\left(f\left(P_{n}\right)\right)\right)<\text{max}\left\{2\varepsilon ,\varepsilon_{J}\right\},$$

where ε and $\varepsilon_{J}$ are sufficiently small, thus, completing the proof. □

In other words, the weighted persistent diagrams of persistent Jones polynomials are stable under small-amplitude or possibly irregular perturbations.

## 5. Applications

The utility of the proposed multi-scale and persistent Jones polynomial models is demonstrated through their application to real-world problems. These models provide robust frameworks for analyzing both local and global structural properties of curves in the 3-space. By leveraging their capacity to encode entanglement complexity features, these models provide new perspectives and tools for understanding protein flexibility, stability, and entanglement.

In this section, we illustrate two important applications of the proposed models. The first application focuses on predicting the B-factor of protein residues, a critical indicator of protein flexibility. This application demonstrates the practical utility of the multi-scale Jones polynomial in processing curve structural data. The second application uses persistent Jones polynomial to analyze the topological features of protein secondary structures, specifically α-helix and β-sheets. These applications demonstrate the versatility and potential of the proposed models to advance computational structural biology.

### 5.1. Multi-scale Jones polynomial for B-factor prediction

B-factors, also known as Debye-Waller factors, measure atomic displacements within protein structures and provide insight into molecular flexibility and stability. Analysis of B-factors enables a deeper understanding of protein dynamics and aids in predicting regions with high structural mobility, which is crucial for understanding protein function and interactions.

To eliminate the influence of irrelevant atomic information and better capture the geometric and topological properties of the protein structure, each amino acid is represented by its $C_{\alpha }$ atom. These $C_{\alpha }$ atoms are sequentially connected to form a $C_{\alpha }$ chain L. Let $C=\left\{c_{0},c_{1},\ldots ,c_{n}\right\}$ denote the set of $C_{\alpha }$ atoms arranged in the sequence of the protein. The $C_{\alpha }$ chain of the protein is considered a disjoint open curve. Segmentation of the $C_{\alpha }$ chain is achieved by cutting the midpoint between each $C_{\alpha }$ atom and its adjacent $C_{\alpha }$ atom, denoted by $P_{n}=\left\{l_{0},l_{1},\ldots ,l_{n}\right\}$. The distance between two curve segments is defined as the distance between the $C_{\alpha }$ atoms contained in the segments, that is, $d\left(l_{i},l_{j}\right)=d\left(c_{i},c_{j}\right)$.

In this study, we select the radius r to range from 4Å to 15Å with a step size of 0.25Å. We set R=(r+1)Å, so the radius R ranges from 5Å to 16Å. In total, the interception range is from 4Å to 16Å. Thus, there is a characteristic matrix for $P_{n}$ of the protein $C_{\alpha }$ chain L,

$$\left(\begin{array}{cccc} JP_{4Å,5Å}^{1}(10) & JP_{4.25Å,5.25Å}^{1}(10) & \cdots & JP_{15Å,16Å}^{1}(10)\\ JP_{4Å,5Å}^{2}(10) & JP_{4.25Å,5.25Å}^{2}(10) & \cdots & JP_{15Å,16Å}^{2}(10)\\ \vdots & \vdots & \ddots & \vdots \\ JP_{4Å,5Å}^{n}(10) & JP_{4.25Å,5.25Å}^{n}(10) & \cdots & JP_{15Å,16Å}^{n}(10) \end{array}\right).$$

This choice is motivated by the fact that the average distance between $C_{\alpha }$ atoms is approximately 3.8Å. The selected radius scheme results in a powerful feature extraction method that provides rich representations of local protein structures. To minimize the influence of overly complex machine learning models, and to emphasize the effectiveness of the multi-scale Jones polynomial and avoid overfitting, we chose to use a Lasso regression model with parameter 0.16 for B-factor prediction.

**Figure 6.**
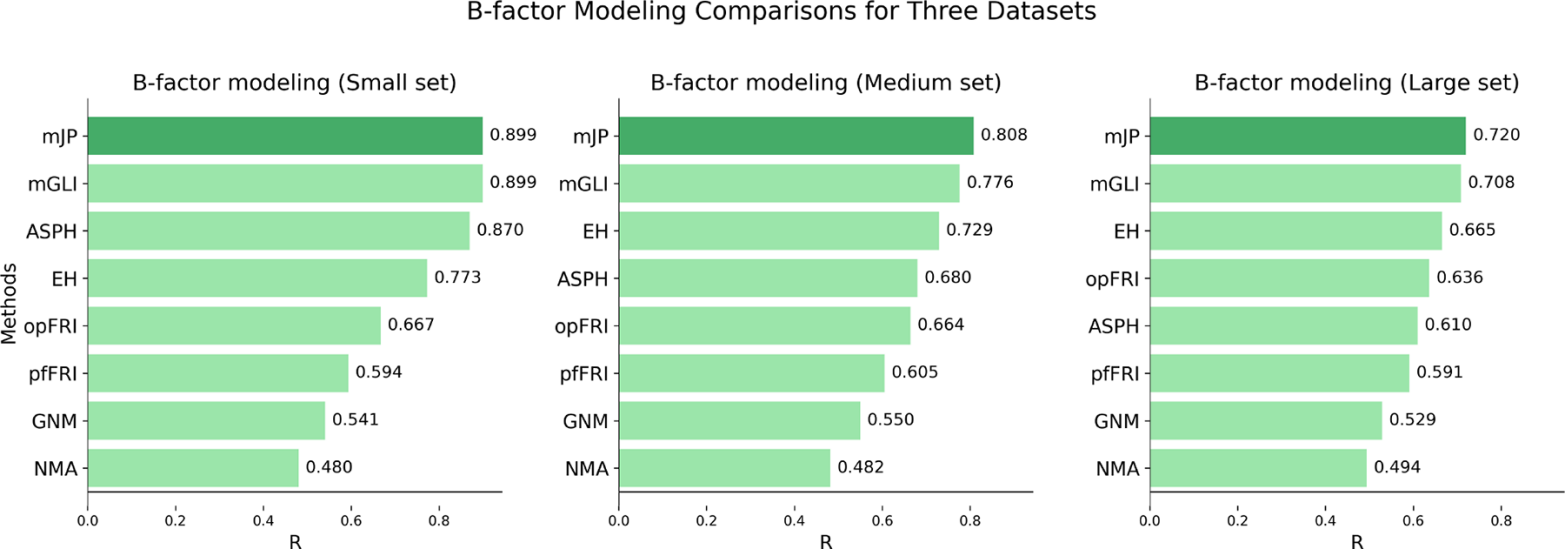
Comparison of B-factor predictions on three protein datasets between our multi-scale Jones polynomial method and other approaches from the literature.

To validate the effectiveness of the multi-scale Jones polynomial in predicting $C_{\alpha }$ atom B factors across proteins of varying sizes, we compared our method with several previous approaches, including mGLI [20], evolutionary homology (EH) [37], atom-specic persistent homology (ASPH) [38], optimal flexibility-rigidity index (opFRI) [39], parameter free flexibility-rigidity index (pfFRI) [39], Gaussian network model (GNM) [30], and normal mode analysis (NMA) [30]. The comparison was performed on three sets of proteins from [30], as shown in Figure 6.

The multi-scale Jones polynomial method achieved average correlation coefficients of 0.899, 0.808, and 0.720 for small, medium, and large protein sets, respectively. Our results on these three datasets outperformed previous methods.

**Figure 7.**
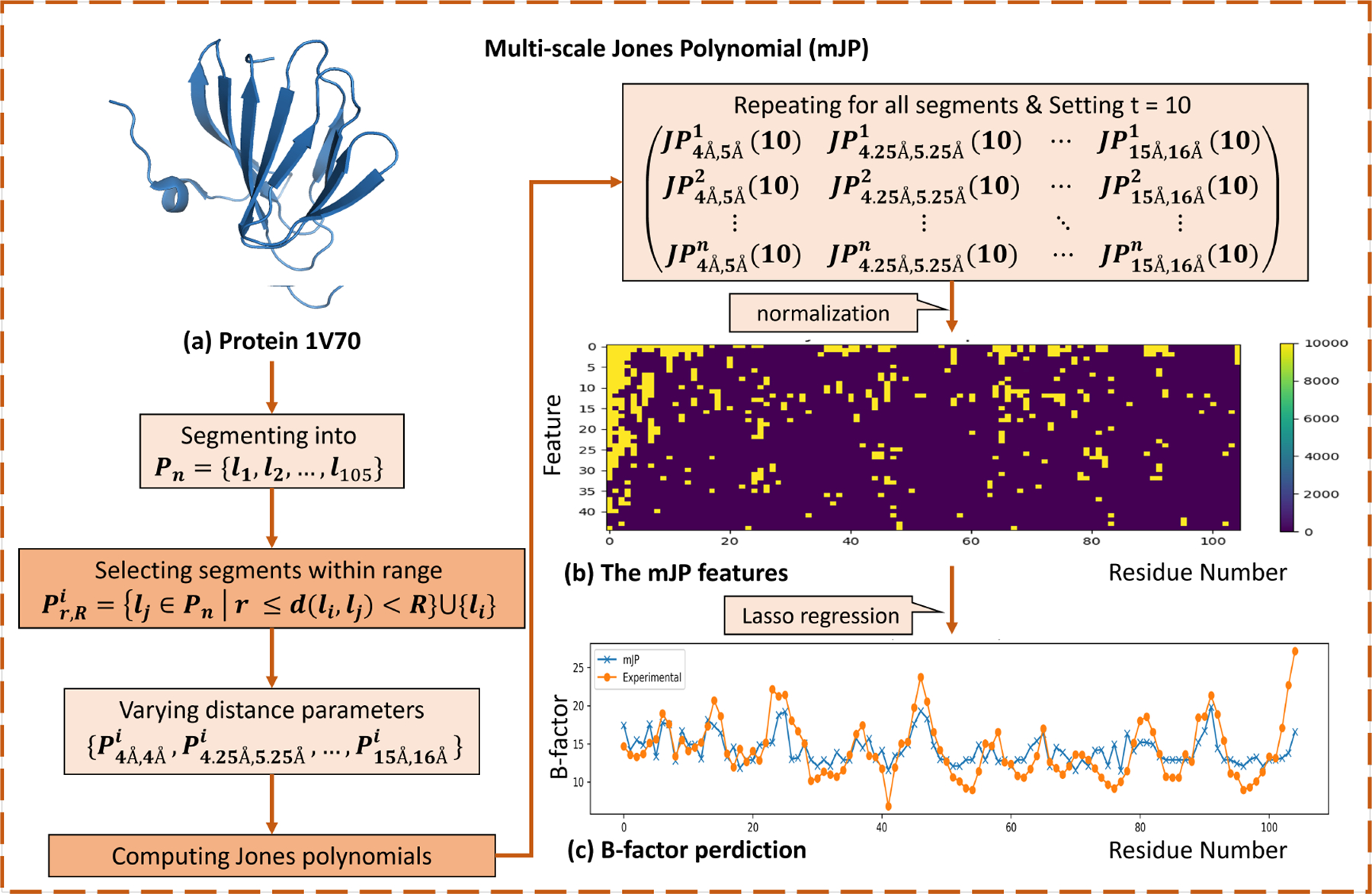
The process of mJP analysis for protein B-factor prediction. (a) The 3D structure of the protein 1V70; (b) The normalized characteristic matrix; (c) A comparison of the predicted B-factors with experimentally determined values.

To further illustrate the performance of the multi-scale Jones polynomial (mJP) analysis, we present a case study involving a potential antibiotic synthesis protein (PDBID: 1V70) containing 105 residues, as shown in Figure 7(a). After processing with the mJP model, as shown in Figure 7, a characteristic matrix is generated. The normalized characteristic matrix is then used as the input to the Lasso regression model, as illustrated in Figure 7(b). The Lasso regression model is used to predict the B-factor of each residue and compare the predicted values with the experimentally determined values, as shown in Figure 7(c).

Compared to traditional B-factor analysis methods, which focus on individual atoms, their spatial positions in the 3-space, and the thermal motion and disorder of atoms within the protein structure, our approach effectively captures the torsional entanglement of the peptide chain at each $C_{\alpha }$ atomic position by incorporating the Jones polynomial. This torsional entanglement of the peptide chain significantly influences the observed B-factor values.

The torsional entanglement of protein peptide chains, captured through the multi-scale Jones polynomial, provides critical insight into the structural and functional dynamics of proteins. By analyzing torsional entanglement, this approach reveals patterns of molecular flexibility and rigidity that allow for an in-depth understanding of protein stability and function. Furthermore, this method improves our ability to model and predict regions of structural mobility, offering potential applications for protein engineering and drug discovery.

### 5.2. Barcodes of persistent Jones polynomial of α-helix and β-sheets

In molecular biology, α-helices and β-sheets are fundamental secondary structures in proteins, stabilized by hydrogen bonding patterns that contribute to the overall stability and function of the protein. Additionally α-helices are typically more rigid than β-sheets. To explore the local structural complexity and stability of these structures, we employed topological analysis using the barcode representation of the persistent Jones polynomial (pJP). Using protein data from the Protein Data Bank (PDB), we demonstrated this approach with examples, including the analysis of an α-helix chain consisting of 19 residues of the protein with PDB ID 1C26. Additionally, we extracted two parallel β-sheets consisting of 16 residues from the protein 2JOX to explore their barcodes representations of the persistent Jones polynomial.

**Figure 8.**
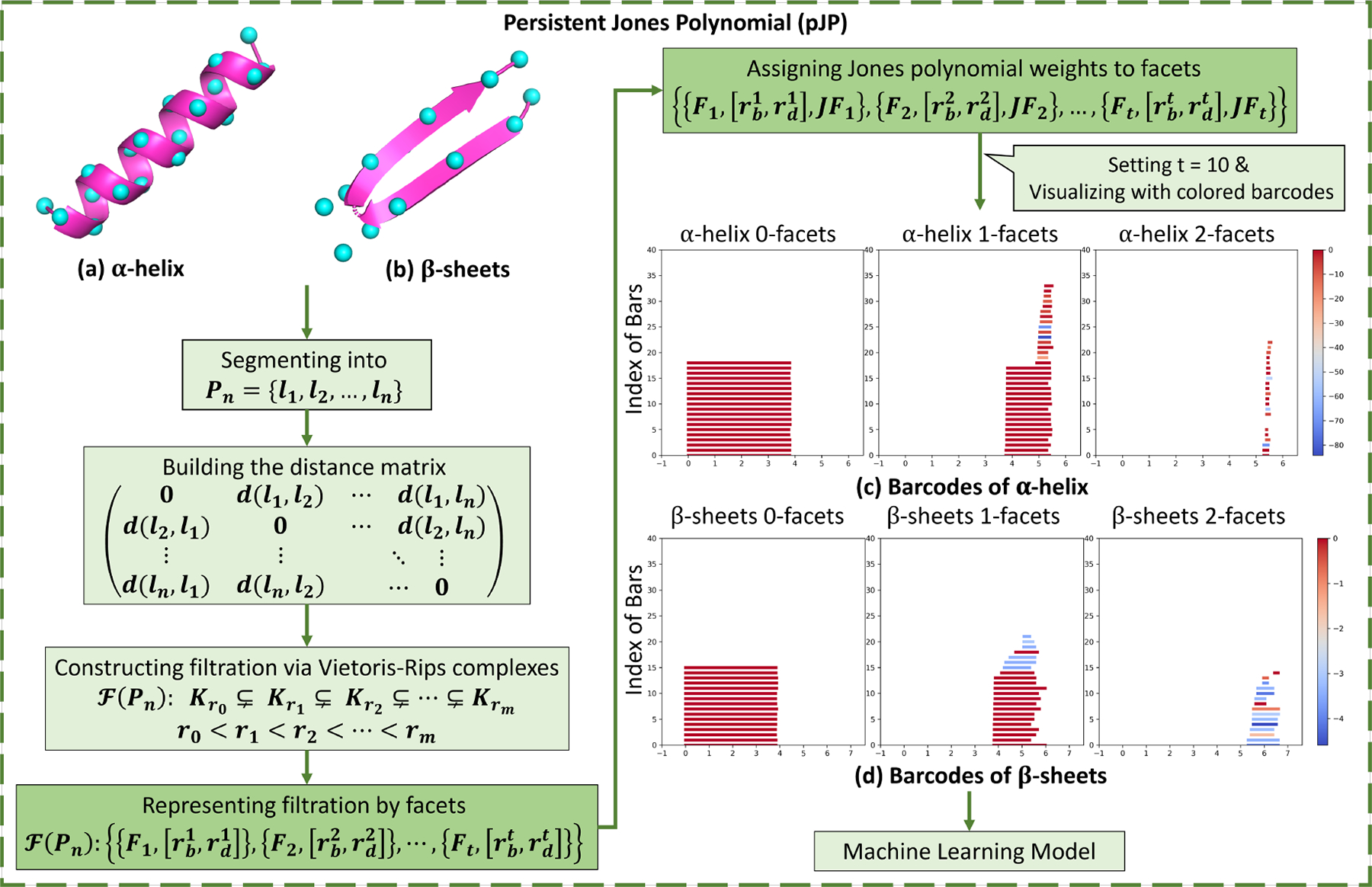
The process of the pJP analysis for the α-helix and β-sheets. (a) (b) The 3D structures of the α-helix and β-sheets. (c) The colored barcodes visualizing the α-helix. (d) The colored barcodes visualizing the β-sheets. The colored barcodes obtained through the pJP model can be applied to machine learning for protein structure analysis.

To eliminate the influence of irrelevant atomic information and better capture the geometric and topological properties of the protein structure, each amino acid is represented by its $C_{\alpha }$ atom, as shown in Figure 8(a) and (b). These $C_{\alpha }$ atoms are sequentially connected to form a $C_{\alpha }$ chain, which is treated as a disjoint open curve. Segmentation of the $C_{\alpha }$ chain is achieved by cutting the midpoint between each $C_{\alpha }$ atom and its adjacent $C_{\alpha }$ atom. The distance between two curve segments is defined as the distance between the $C_{\alpha }$ atoms contained within these segments.

The process of the pJP model is illustrated in Figure 8, using colored barcodes to represent the α-helix and β-sheets structures.

Figure 8(c) represents the barcodes corresponding to the α-helix. In the 0-facets panel, there are 19 bars with Jones polynomial weights of 0. Each bar has a length of approximately 3.8Å, which is the average distance between two $C_{\alpha }$ atoms. Additionally, the 1-facets panel contains 18 bars with similar birth times and life-spans, starting around 3.8Å and persisting until approximately 5.4Å, each with a Jones polynomial weight of 0. These bars correspond to facets formed by two adjacent $C_{\alpha }$ atoms. Moreover, 16 short-lived bars represent facets formed by two nonadjacent $C_{\alpha }$ atoms. As shown in the 2-facets panel, the bars capture the persistence of facets formed by three $C_{\alpha }$ atoms, along with the complexity of entanglement of the corresponding polyline system.

Figure 8(d) represents the barcodes corresponding to the β-sheets. Similar to the α-helix case, the segmentation and distance definitions are consistent. The 0-facets panel includes 16 0-facets bars, indicating the presence of 16 $C_{\alpha }$ atoms. In the 1-facets panel, there are 14 bars for facets formed by two adjacent $C_{\alpha }$ atoms and 8 for facets formed by nonadjacent $C_{\alpha }$ atoms. The bars in the 2-facets panel represent facets formed by three $C_{\alpha }$ atoms and provide information about the complexity of entanglement. The longer lifespans of the bars in the 2-facets panel of the β-sheets compared to the α-helix suggest that the $C_{\alpha }$ atoms in the β-sheets are more spatially dispersed.

The color of the barcodes reflects the value of the Jones polynomial weight, which indicates the difference of the torsional entanglement of the set of curve segments in the system. It is important to note that the color gradient, whether it tends to red or blue, does not imply a higher or lower degree of entanglement complexity in the represented set of curve segments. Rather, a greater color difference between two bars indicates a greater difference between the sets of curve segments they represent.

From the color bars in Figures 8 (c) and (d), it can be observed that the Jones polynomial weight for the α-helix ranges from −86 to 0, while for the β-sheets it ranges from −6 to 0. This indicates a greater variability in the sets of curve segments represented by the facets within the α-helix compared to the β-sheets, suggesting that the α-helix exhibits more complex entanglement, as observed.

The persistent Jones polynomial effectively captures the torsional entanglement of secondary structures, such as α-helices and β-sheets, within protein peptide chains. This torsional entanglement plays a critical role in the analysis of protein structure and function as it provides insight beyond atomic positions alone. By incorporating the Jones polynomial, our approach reveals important topological characteristics that are important for protein stability and functionality.

## 6. Concluding remarks

In this study, the selection of the segmentation $P_{n}=\left\{l_{1},l_{2},\ldots ,l_{n}\right\}$ for a collection of curves L is crucial to capture the topological and geometric characteristics of the curve structure L. The segmentation serves as the basis for defining and calculating both the multi-scale analysis of the Jones polynomial and the persistent Jones polynomial. First, the outcomes of these two models depend not only on the spatial positions of the segments but also on their relative lengths in relation to the entire curve. As the segment length approaches zero, the results of the models tend toward triviality. Similarly, when the segment extends to cover the entire curve, the models recover global information. In both of these cases, the models cannot extract meaningful local information for spatial data. Thus, the choice of segmentation depends on the specific application.

Knot theory has traditionally focused on global invariants, but real-world applications often require local structural insights. Classical knot invariants mainly reflect global topology and fail to capture crucial local structural details in applications like molecular biology and highway crossing design. To address this gap, localized versions of invariants such as the multi-scale Jones polynomials and persistent Jones polynomials have been developed. These localized models decompose global invariants for analyzing local topology in the context of the entire structure, promoting the application of KDA or CDA in systems where both global and local structures matter.

The stability of the model is crucial for practical applications. In real-world data, noise and minor perturbations pose challenges. Stability ensures that minor input changes do not cause disproportionate changes in the calculated invariants. It is critical in biological or physical contexts. For multi-scale Jones polynomial and persistent Jones polynomial models, we demonstrate stability under small perturbations. Minor adjustments in the collection L result in slight modifications to characteristic matrices and barcodes or diagrams. This stability makes the models reliable for structural topology and is applicable in KDA or CDA for real-world data.

The torsional entanglement of protein peptide chains, captured through both multi-scale and persistent Jones polynomials, provides crucial insights into the structure and function of proteins. By analyzing torsional entanglement, this approach reveals patterns of molecular flexibility and rigidity that allow a detailed understanding of protein structure and function. Furthermore, this method improves our capacity to model and predict regions of structural mobility and reactivity, offering valuable implications for enzyme kinetics and protein engineering.

In Section 5.2 of this manuscript, the barcodes of the persistent Jones polynomial represent the birth, death, and lifespan of the facets in the complexes under filtration. In contrast, persistent homology barcodes capture the changes in the homology classes of the complexes during filtration, i.e., the changes in the generators of the homology groups of the complexes (the number of generators corresponds to the Betti number). Despite these differences, there are important similarities between the two concepts. Both are based on filtration and provide insight into data characteristics by examining the evolution of the complexes during filtration. In addition, the bars in the barcodes of the persistent Jones polynomial represent facets formed by subsets of curve segments from the segmentation $P_{n}=\left\{l_{1},l_{2},\ldots ,l_{n}\right\}$ of the collection of curves L. Therefore, these bars are constructed based on the distance conditions between the curve segments in the segmentation $P_{n}$. Similarly, in the case of point cloud data, persistent homology barcodes are also constructed according to the distance conditions of the points in the point cloud data.

The multi-scale Gauss link integral model [20] and the present multi-scale Jones polynomial and persistent Jones polynomial models represent solid advances in computational geometric topology. These approaches have great potential for real-world applications when combined with machine learning and artificial intelligence.
